# 

**Published:** 1858-09

**Authors:** 


NORTH AMERICAN

### MEDICO-CHIRURGICAL REVIEW.

Vol. II.] SEPTEMBER, 1858. [No. 5.

### CONTENTS.

### ANALYTICAL AND CRITICAL REVIEWS.

Art. Page

I.—Urinary Deposits; their Diagnosis, Pathology, and Therapeutical Indications. By Golding Bird, M.D. . . . . . 785

II.—Of Nature and Art in the Cure of Disease. By Sir John Forbes, M.D. . . . . . . . . . . . . . 800

III.—The Institutes of Medicine. By Martyn Paine, M.D. . . . 807

IV.—A Manual of Psychological Medicine; Including the History, Nosology, Description, Statistics, Diagnosis, Pathology, and Treatment of Insanity. By John Charles Bucknill, M.D., and Daniel H. Tuke, M.D. . . . . . . 826

V.—Outlines of a Course of Lectures on the Principles and Practice of Surgery. By E. Geddings, M.D. . . . . . 843

VI.—Evil Results of Over-Feeding Cattle. By Frederick James Gant, M.R.C.S. . . . . . . . . . . . . 846

VII.—Elements of Inorganic Chemistry. By Thomas Graham, F.R.S.L. and E. . . . . . . . . . . . . . 848

VIII.—Handbuch der Praktischen Chirurgie für Artze und Wundärtze. By Victor Von Bruns, M.D. . . . . . . . 848

IX.—A Treatise on Rheumatic Gout. By Robert Adams, M.D. . . 850

### ORIGINAL COMMUNICATIONS.

I.—A Case presenting Choreic Symptoms, Terminating in Death. By O. C. Gibbs, M.D. . . . . 851

II.—Phenomena of the Capillary Circulation. By Austin Flint, Jr., M.D. . . . . . . . . . . 861

III.—Proceedings of the Pathological Society of Philadelphia. . . 867

### AMERICAN PROGRESS OF MEDICAL SCIENCE.

Report on Materia Medica and Therapeutics. By John Bell, M.D. Part I. . . . . . . . . . . . . . . . . 910

### BI-MONTHLY FOREIGN ABSTRACTS.

#### SURGERY.

I.—A New Method of Amputation. By M. Maisonneuve. . . 947

II.—On the Scooping out of Bones as a means of preserving their Shape and Functions. By Prof. Sédillot. . . . . . . 948

ART. PAGE
III.—Observations on Hereditary Syphilis of the Bones and Cartilages. By Dr. SIGMUND. . . . . . . . . . 949

OBSTETRICS AND DISEASES OF WOMEN AND CHILDREN.
IV.—On the Pathogeny of Hydrorrhœa of Pregnant Women. By Prof. C. BRAUN. . . . . . . . . . . . 950
V.—On Amputation of the Neck of the Uterus in Prolapsus of this Organ. By C. MAYER, M.D. . . . . . . . . . 951
VI.—On Linear Écrasement of the Cervix Uteri. By Dr. BRESLAU. . 952
VII.—Treatment of Engorgement of the Cervix Uteri with Ointment of the Chloroiodide of Mercury. By M. CH. BERNARD. . . 952
VIII.—Thrombosis of the Cerebral Sinuses in Children. By Dr. GERHARDT. 954

BI-MONTHLY PERISCOPE.

SURGERY.
Excision of Bones and Joints. . . . . . . . . . . . 955

PRACTICE OF MEDICINE.
Observations on the Use of the Shower-Bath in Delirium Tremens. . . 960

OBSTETRICS.
1. On Menstruation during Pregnancy. . . . . . . . . 965
2. Observations on the Beneficial Effects of Pepsin in the Obstinate Vomiting of Pregnancy. . . . . . . . . 965
3. On the Carbonic Acid Gas Douche in Uterine Disease . . . . 966
4. Laudanum Dressings in Painful Affections of the Uterus. . . . 967

EDITORS' TABLE.—Changes and Appointments in Medical Schools. . 969
Charleston Medical and Surgical Journal. . . . . . 969
Shelby Medical College. . . . . . . . . 969
Triplets. . . . . . . . . . . . . . 969
New Medical Journals. . . . . . . . . . 970
Philadelphia Hospital, Blockley. . . . . . . . 970
Naval Appointments. . . . . . . . . . . 970
Army Appointments. . . . . . . . . . . 970
Corn as an Antiperiodic. . . . . . . . . . 970
Chair of Chemistry in the University of Edinburgh. . . . 971
Sir Benjamin Brodie. . . . . . . . . . . 971
The Bréant Prize. . . . . . . . . . . . 971
Novel Cure for Phthisis, and a New Operation of Castration. . . 972
The College of Spirits in Paris . . . . . . . . 972
Monuments to Jenner. . . . . . . . . . . 974
To Correspondents. . . . . . . . . . . . 974
Notice to Foreign Medical Journals. . . . . . . . 974

NECROLOGICAL NOTICES.—The Late Sir Philip Crampton, M.D. . . 974
Dr. John Snow. . . . . . . . . . . . . 975
Professor Busch. . . . . . . . . . . . 975

BI-MONTHLY BIBLIOGRAPHICAL RECORD. . . . . . . . . 976

